# The novel long non-coding RNA TALNEC2, regulates tumor cell growth and the stemness and radiation response of glioma stem cells

**DOI:** 10.18632/oncotarget.15991

**Published:** 2017-03-07

**Authors:** Shlomit Brodie, Hae Kyung Lee, Wei Jiang, Simona Cazacu, Cunli Xiang, Laila M Poisson, Indrani Datta, Steve Kalkanis, Doron Ginsberg, Chaya Brodie

**Affiliations:** ^1^ Everard and Mina Goodman Faculty of Life Sciences, Bar-Ilan University, Ramat-Gan, Israel; ^2^ Davidson Laboratory of Cell Signaling and Tumorigenesis, Hermelin Brain Tumor Center, Department of Neurosurgery, Detroit, MI, USA; ^3^ Department of Public Health Sciences, Center for Bioinformatics, Henry Ford Hospital, Detroit, MI, USA

**Keywords:** TALNEC2, long non-cording RNAs, glioblastoma, glioma stem cells, mesenchymal transformation

## Abstract

Despite advances in novel therapeutic approaches for the treatment of glioblastoma (GBM), the median survival of 12-14 months has not changed significantly. Therefore, there is an imperative need to identify molecular mechanisms that play a role in patient survival. Here, we analyzed the expression and functions of a novel lncRNA, TALNEC2 that was identified using RNA seq of E2F1-regulated lncRNAs. TALNEC2 was localized to the cytosol and its expression was E2F1-regulated and cell-cycle dependent. TALNEC2 was highly expressed in GBM with poor prognosis, in GBM specimens derived from short-term survivors and in glioma cells and glioma stem cells (GSCs). Silencing of TALNEC2 inhibited cell proliferation and arrested the cells in the G1\S phase of the cell cycle in various cancer cell lines. In addition, silencing of TALNEC2 decreased the self-renewal and mesenchymal transformation of GSCs, increased sensitivity of these cells to radiation and prolonged survival of mice bearing GSC-derived xenografts. Using miRNA array analysis, we identified specific miRNAs that were altered in the silenced cells that were associated with cell-cycle progression, proliferation and mesenchymal transformation. Two of the downregulated miRNAs, miR-21 and miR-191, mediated some of TALNEC2 effects on the stemness and mesenchymal transformation of GSCs. In conclusion, we identified a novel E2F1-regulated lncRNA that is highly expressed in GBM and in tumors from patients of short-term survival. The expression of TALNEC2 is associated with the increased tumorigenic potential of GSCs and their resistance to radiation. We conclude that TALNEC2 is an attractive therapeutic target for the treatment of GBM.

## INTRODUCTION

The human genome expresses tens of thousands of long non-coding RNAs (lncRNAs), which are >200 bases in length but lack significant open reading frames [1]. lncRNAs exhibit diverse transcriptional patterns that have important functions in various cellular processes in both physiological and pathological conditions and exhibit tissue specificity. Many lncRNAs are frequently aberrantly expressed in various human cancers, with potential roles in both oncogenic and tumor suppressive pathways [2–5]. Of note, the promoters of many lncRNAs are bound and regulated by transcription factors known to influence mRNA transcription, including cancer-related transcription factors, such as p53 [6], Myc [7, 8] and E2F [9–11]. LncRNAs can be expressed either in intergenic, intron regions or in overlapping or antisense locations adjacent to protein-coding genes. New lncRNAs rapidly continue to be identified and many of them are localized in the cytoplasm rather than in the nucleus [12].

Glioblastoma (GBM) is a heterogeneous neoplasm with a small percentage of long-term survivors [13]. Despite aggressive surgical resection and advances in radiotherapy and chemotherapy, the median survival for patients with GBM is around 12–14 months [14]. Recent studies have identified various molecular subtypes of GBM that exhibit different phenotypes and have important clinical and prognostic implications [15, 16]. Limitations to therapy include the distinctly infiltrative nature of the tumors, which prevents complete resection [17] and the high resistance to radio- and chemotherapy of residual tumor cells and glioma stem-like cells (GSC) [18].

GSCs are a small subpopulation of self-renewing and tumorigenic cancer stem cells that have been implicated in tumor infiltration, resistance to conventional therapies and tumor recurrence [18, 19]. Therefore, understanding the mechanisms associated with the stemness and oncogenic features of these cells is essential for the development of therapeutic approaches that can eradicate GSCs and may provide the basis for the development of novel therapeutic approaches for the treatment of GBM. GSCs can generate the different cell types that constitute the tumor and neurosphere-derived xenografts have been shown to recapitulate the molecular and cellular profiles of the parental tumors [20, 21].

The expression of lncRNAs has been recently characterized in GBM [22, 23] and lncRNA signatures and profiles in various GBM subtypes [24] and in association with patient prognosis [25, 26] have been identified. Moreover, the roles of specific lncRNAs such as H19 [27] and MALAT1 [28] have been described in various functions of glioma cells [29, 30], whereas the function of lncRNAs in the functions of GSCs is just beginning to be understood.

In this study, we describe the identification of a novel E2F-regulated lncRNA that regulates tumor cell growth, stemness of glioma stem cells and their radiation sensitivity. Moreover, we demonstrated that this lncRNA was increased in GBM compared to low-grade glioma and normal brain tissues and was highly expressed in GBM specimens derived from short-term survival patients. Based on its location in chromosome 2 and tumor-associated function, we named this lncRNA, TALNEC2 (Tumor associated lncRNA expressed in chromosome 2).

## RESULTS

### Identification of the novel lncRNA TALNEC2

**Figure 1:**
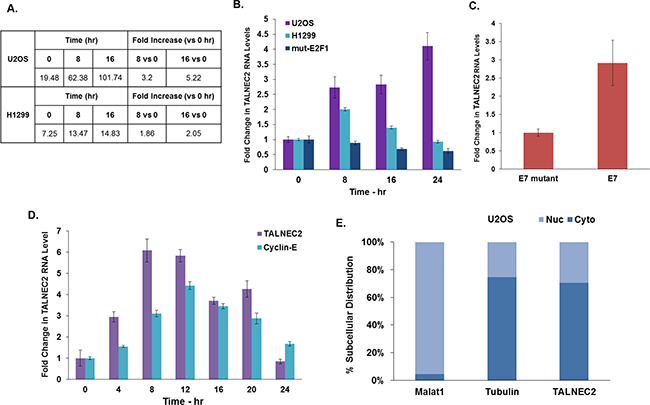
Characterization of TALNEC2 expression and subcellular localization RNA Seq. results for TALNEC2 are shown as fold of induction after addition of OHT for 8 and 16 h compared to untreated cells **(A)**. U2OS and H1299 cells expressing ER-E2F1 and U2OS cells expressing mutated ER-E2F1 were treated with OHT for the indicated time points (in hours). RNA was extracted and TALNEC2 RNA levels were determined by real-time PCR and normalized to GAPDH levels. Real-time PCR experiments were performed in triplicates **(B)**. WI38 cells were infected with a retrovirus vector expressing either wild-type E7 (E7) or an RB-binding–deficient mutant of E7 (E7-mutant). RNA was extracted and TALNEC2 RNA levels were determined by Real-time PCR and normalized to GAPDH levels. Real-time PCR experiments were performed in duplicates **(C)**. WI38 cells were growth arrested by serum deprivation (48 hours in 0.1% serum) and then allowed to resume growth by serum addition (to a final concentration of 15%) for the indicated times. RNA was extracted and levels of CCNE1 and TALNEC2 RNA levels were determined by Real-time PCR and normalized to GAPDH levels. CCNE1 is a known E2F-regulated gene serving as a positive control for cell cycle-dependent gene expression. Real-time PCR experiments were performed in triplicates **(D)**. RNA was extracted from the nuclear and cytoplasmic fractions of the U2OS cells as described in the methods and the levels of nuclear control transcript (MALAT1), cytoplasmic control transcript (GAPDH or tubulin), and TALNEC2 were determined by RT-PCR in the nuclear and cytoplasmic fractions. The bar graphs present the percentage of nuclear and cytoplasmic RNAs out of the total cell RNA. Real-time PCR experiments were performed in triplicate **(E)**. The results are representative of three different experiments that gave similar results.

TALNEC2 (Tumor Associated Long Non-coding RNA Expressed on Chromosome 2) is a 1,407 bp long intergenic lncRNA (localized between genes in a non-protein-coding region) that is located on the reverse strand and consists of three exons. TALNEC2 (also termed ENST00000295549 [Ensembl] and LINC01116 [RefSeq]) was originally identified as a putative E2F1-regulated lncRNA [10] through a deep-sequencing analysis preformed using U2OS human osteosarcoma cells and H1299 human non-small-cell lung carcinoma cells expressing a conditionally overexpressed E2F1 vector (Figure 1A). qRT-PCR analysis further validated the expression data obtained from the RNA-sequencing in U2OS and H1299 cells expressing the conditionally active E2F1 (Figure 1B). Activation of mutated E2F1 that does not bind DNA did not significantly affect TALNEC2 expression, demonstrating that the E2F1-induced upregulation of TALNEC2 was a result of the transcriptional activity of E2F1 (Figure 1B).

We next tested whether TALNEC2 RNA levels are induced in response to activation of endogenous E2Fs. To activate the endogenous E2Fs we employed the E7 HPV16 protein, which disrupts the pRB/E2F complexes. WI38 human fibroblasts expressing wild type E7 exhibited an increase in the RNA levels of TALNEC2 compared to cells expressing the mutant E7, which does not bind pRB (Figure 1C). These results indicate that in addition to being regulated by ectopic E2F1, TALNEC2 was also regulated by the endogenous E2Fs.

Furthermore, TALNEC2 RNA levels increased in response to activation of ectopic E2F1, even in the presence of CHX, indicating that de novo protein synthesis was not required for E2F1-mediated upregulation of TALNEC2 and suggesting that it is a direct E2F1 target (Supplementary Figure 1A). Moreover, analysis of the genomic sequence upstream of human TALNEC2 transcription start site identified three putative E2F binding sites. Indeed E2F1 was found to be bound to this putative promoter as published by ChIP-seq analysis performed as part of the encode project displayed as tracks in the UCSC Genome Browser (Supplementary Figure 1B). Expression levels of TALNEC2 across 54 tissues was explored using the Genotype-Tissue Expression (GTEx) Consortium data portal (http://www.gtexportal.org/home/gene/AC017048.3#geneExpression, last accessed 4/20/16) [31], and was found to be variable among tissues with low expression in the brain (Supplementary Figure 2A). In addition, there appears to be variable expression of TALNEC2 in various adult and fetal normal cells (Supplementary Figure 2B).

### TALNEC2 is a cell cycle-regulated cytosolic lncRNA

To further characterize TALNEC2, we examined its expression during cell-cycle progression. For these studies, we employed cells that were growth arrested by serum deprivation and were later allowed to resume growth by serum addition. Changes in cell cycle progression were monitored using FACS analysis [10]. TALNEC2 exhibited a “bell-shaped” pattern of expression during cell cycle progression with its peak as cells entered the G1/S transition. This pattern of expression is similar to Cyclin-E, a known E2F- and cell cycle-regulated gene (Figure 1D).

We then analyzed the subcellular localization of TALNEC2 in three different cell lines; U2OS human osteosarcoma cells (Figure 1E), MCF-7 breast cancer cells (Supplementary Figure 3A) and H1299 human non-small-cell lung carcinoma cells (Supplementary Figure 3B). TALNEC2 RNA was enriched in the cytoplasm of these cells, similar to the cytoplasmic control transcripts (GAPDH and tubulin), as opposed to the nuclear control (the lncRNA MALAT1) [28]. This subcellular localization was similar in all three cell lines examined.

### Biological functions of TALNEC2

**Figure 2:**
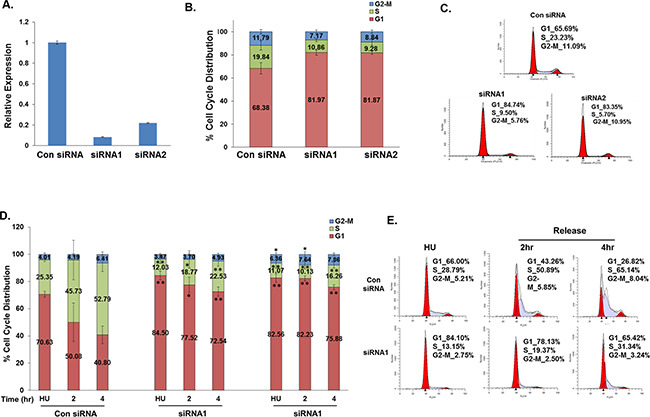
Silencing of TALNEC2 arrests cells in the G1 phase of the cell cycle MCF-7 cells were transfected with either a nonspecific siRNA (Con siRNA, 100nM ) or siRNAs directed against TALNEC2 (siRNA1 (25nM), siRNA2 (100nM)). Cells were harvested 72 hours post transfection **(A, B, C)**. RNA was extracted and TALNEC2 RNA levels were determined by real-time PCR and normalized to GAPDH levels. Real-time PCR experiments were performed in triplicates **(A)**. Cells were analyzed by FACS using propidium-iodide (PI) protocol. Percentages of cells in G1, S, and G2/M cell-cycle phases are shown **(B, C)**. The results are either representative **(C)** or average of four independent experiments **(B)**. *P<0.05, **P<0.01 (two-tailed students t-test). MCF-7 or H1299 cells were transfected with either a nonspecific siRNA (Con siRNA, 100nM) or siRNAs directed against TALNEC2 (siRNA1 (25nM), siRNA2 (100nM). Next, cells were incubated with hydroxyurea (HU, 2mM) for 18 h. 72 h post transfection cells were harvested or allowed to resume growth by fresh media wash and growth in the fresh media for times indicated. Cells were analyzed by FACS using propidium-iodide (PI) protocol **(D, E)**. Percentages of cells in G1, S, and G2-M cell-cycle phases are depicted. **(D, E)**. Average FACS analysis for MCF-7 cells is for four independent experiments **(D)**. *P<0.05, **P<0.01 (two-tailed students t-test). For H1299 cells, the results are representative of three independent experiments **(E)**.

TALNEC2 functions were analyzed in the proliferation, cell-cycle progression and apoptosis of different cancer cell lines. Silencing of TALNEC2 in MCF-7 breast cancer cells by two specific siRNAs (Figure 2A) resulted in a significant increase in the number of cells in the G1 phase of the cell cycle and a concomitant reduction in the number of cells in S phase suggesting that TALNEC2 may have a role in G1 exit (Figures 2B, 2C). Similar results were also observed in the H1299 cells (Supplementary Figure 4A, 4B, 4C). To analyze the potential role of TALNEC2 in G1 exit, MCF-7 cells were synchronized with hydroxyurea (HU), which inhibits ribonucleotide reductase thereby synchronizing cells in the G1/S transition. The cells were then released from the hydroxyurea block and their re-entry into the cell cycle was monitored. Hydroxyurea-treated cells were arrested in G1 and silencing of TALNEC2 enhanced this arrest. Importantly, as cells were released from the hydroxyurea block, fewer cells exited G1 and transitioned into S phase in TALNEC2-silenced cells (Figures 2D, Supplementary Figure 5A, 5B). Similar results were also observed in the H1299 cells (Figures 2E, Supplementary Figure 5C). These results indicate that silencing TALNEC2 inhibited G1 exit and cell-cycle progression.

**Figure 3:**
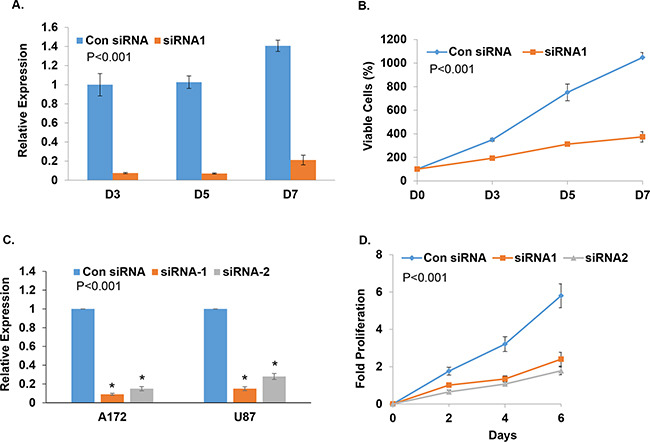
Silencing of TALNEC2 inhibits cell proliferation MCF-7 cells were transfected with either a nonspecific siRNA (Con siRNA, 25nM ) or siRNA directed against TALNEC2 (siRNA1, 25nM). Cells were harvested at the indicated days post transfection **(A, B)**. RNA was extracted and TALNEC2 RNA levels were determined by Real-time PCR and normalized to GAPDH levels. Real-time PCR experiments were performed in triplicates **(A)**. Cell proliferation was measured by MTT assay **(B)**. U87 and A172 glioma cells were transfected with either a nonspecific siRNA (Con siRNA) or siRNAs directed against TALNEC2 (siRNA1, siRNA2). Levels of TALNEC2 were determined after 3 days using RT-PCR **(C)** and cell proliferation was determined for the U87 cells at the indicated time points using MTT **(D)**. The results are representative of three different experiments that gave similar results.*P<0.001 **P< 0.01.

To test the effects of TALNEC2 on cell proliferation and viability, we employed the MTT assay using both the MCF-7 and the glioma cells U87 and A172. The different cell lines were silenced for TALNEC2 and the numbers of viable cells were assessed for up to seven days. Significant silencing of TALNEC2 persisted for at least 7 days after transient transfection for all the cell lines examined (Figures 3A, Supplementary Figure 6A, 6C). Silencing of TALNEC2 inhibited the growth of MCF-7 cells by up to 66% (Figures 3B, Supplementary Figures 6B, 6D), and this inhibition was not associated with increased cell death. Silencing of TALNEC2 in glioma cell lines U87 and A172 (Figure 3C) also decreased cell proliferation, which started after 24 h of silencing (Figures 3D, Supplementary Figure 6E).

### TALNEC2 expression in GBM, glioma cells and GSCs

**Figure 4:**
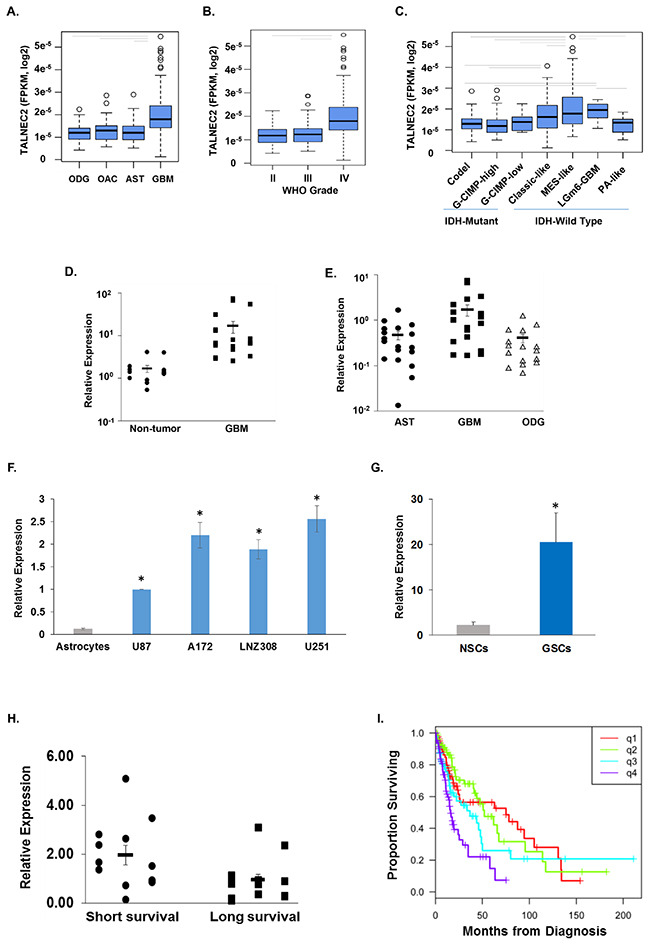
Expression of TALNEC2 in GBM, glioma cell lines and GSCs Expression of TALNEC2 in glioma tissues was determined from RNA-sequencing data from The Cancer Genome Atlas project. The distribution of expression is shown by boxplots for histology **(A)**, WHO grade **(B)**, and supervised IDH-methylation classes **(C)**. Average expression was compared by ANOVA (P<0.0001) with follow-up t-tests (P<0.05, grey bars). Total RNA was extracted from normal brains (Non-tumor), astrocytoma (AST), oligodendroglioma (ODG) and GBM specimens and the expression of TALNEC2 was determined using real-time PCR **(D, E)**. Results are normalized relative to the levels of S12 mRNA and are presented relative to a reference sample as dot-plots around the estimate of the mean and SEM bars. GBM has higher TALNEC2 expression, on average, than non-tumor brain, AST, or ODG (P<0.01). The mean expression of TALNEC2, measured using real-time PCR, in glioma cell lines relative to human astrocytes **(F)** is shown by barplots with SEM bars (student test P<0.001 indicated by *). Twelve different primary GSCs, generated from GBM specimens, show increased mean expression of TALNEC2 (by RT-PCR), relative to human NSCs (**G**, P<0.001). GBM specimens obtained from sort-term survivors (<9 months, n=13) have higher mean TALNEC2 expression compared to long-term survivors (>36 months, n=12; P< 0.01, **H**). Kaplan-Meier survival estimates of overall survival are plotted for patients grouped by TALNEC2 expression quartiles (q1=lowest expression; log-rank p= 0.000064, I). The results are presented as the mean values ± SD. Data were analyzed using analysis of variance or a Student's t-test.

We further focused on the expression and function of TALNEC2 in glioma. We first analyzed the expression of TALNEC2 in glioma specimens using TCGA data. Expression data for TALNEC2 (LINC00116) was obtained for 341 primary glioma samples profiled by RNA-sequencing through TCGA (205 LGG; 136 GBM). There was a significant difference between the mean TALNEC2 expression by histology (ANOVA, P<0.0001), with GBM showing higher mean expression than the other groups (Figure 4A, t-tests, P<0.0001). This was also reflected in a difference by WHO Grade (ANOVA, P<0.0001); with Grade IV tumors showing significantly higher mean expression (Figure 4B, t-tests, P<0.0001). Going across histological boundaries, TALNEC2 expression was highest among IDHwt tumors (‘molecular glioma’) compared to IDH-mutant tumors (t-test, P<0.0001). Separation of tumors into the seven IDH-methylation subclasses showed that mean expression differences persisted (ANOVA, P<0.0001); with the aggressive IDHwt groups (Classic-like, Mesenchymal-like, LGm6-GBM) showing higher mean levels than the less-aggressive IDHmut groups (Figure 4C, CODEL, GCIMP-High; t-tests p-values [<0.0001 – 0.0203]). The GCMIP-low group harbors an IDH mutation but demonstrates an aggressive phenotype. Here we found the GCMIP-low group had lower mean expression than the mesenchymal-like tumors (t-test, P=0.0102), but there was insufficient evidence to separate it from the other classes. The PA-like group was molecularly similar to the LGm6-GBM class, but had clinical outcomes similar to the Grade I pilocytic astrocytoma. We also observed that the LGm6-GBM class had higher mean expression of TALNEC2 than the PA-like group (t-test, P=0.0427). Altogether, these results indicate that higher expression of TALNEC2 is found in tumors with poorer prognosis.

We then analyzed the expression of TALNEC2 in GBM and glioma cell lines. We first examined the expression of TALNEC2 in normal brain and GBM specimens using qRT-PCR. We found that the expression of TALNEC2 mRNA was significantly higher in GBM specimens compared with that of normal brains (Figure 4D, P<0.001) and that the expression of TALNEC2 was increased in GBM compared to anaplastic astrocytomas and oligodendrogliomas (Figure 4E, P< 0.001). The expression of TANEC2 was also examined in the glioma cell lines A172, U251, U87, T98G and LNZ308, and these cell lines expressed higher levels of TALNEC2 compared to human astrocytes (Figure 4F, P<0.001).

GBM contain a small population of GSCs, which have been implicated in the resistance to therapy and recurrence of these tumors [18, 19]. Using 12 lines of GSCs that were generated from fresh GBM specimens as described [32, 33], we found that the expression of TALNEC2 in these cells was significantly higher than that of human neural stem cells (NSCs) (Figure 4G, P<0.001).

Similar to its expression in glioma cells, TALNEC2 was also expressed in most cancer cell lines examined (Supplementary Figures S7).

### TALNEC2 expression is increased in GBM specimens from short-term survival patients

Prognosis in GBM patients has been associated with various factors including age, tumor size, extent of tumor resection of >98%, tumor location and O6-methylguanine methyltransferase (MGMT) promoter methylation [34]. In addition, deregulation of non-coding RNA expression has been also implicated in patient prognosis [23]. We therefore examined whether the expression of TALNEC2 was associated with patient survival. For these experiments we employed 25 samples of GBM specimens derived from patients of short (< 9 months, n=13) or long (> 3 years, n=12) survival. Using RT-PCR we found that the expression of TALNEC2 was significantly (P<0.01) higher in the GBM specimens derived from the patients of short-term survival (Figure 4H). Thus, TALNEC2 can act as a prognostic marker in GBM.

Analysis of overall survival across glioma, ignoring all other factors contributing to survival, demonstrated that increasing TALNEC2 expression (from Q1 to Q4) is associated with increased risk of death (Figure 4I, i.e., shorter survival, log-rank P=0.000064).

### TALNEC2 regulates the stemness and mesenchymal transformation of GSCs and modulates their response to radiation

**Figure 5:**
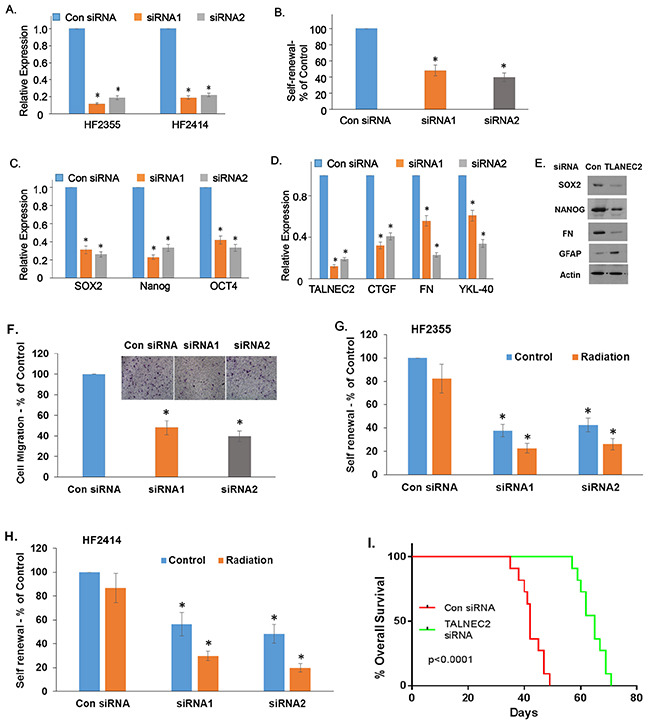
TALNEC2 regulates GSC stemness, mesenchymal transformation and response to radiation GSCs transfected with a control or TALNEC2 siRNAs were analyzed for the expression of TALNEC2 **(A)**. The HF2355 GSCs were then plated at 100 cells/well in 96-well plates and the number of neurospheres per well was quantified after 14 days **(B)**. P < 0.0001. The expression of the stemness markers, NANOG, SOX2 and OCT4 **(C)**, and the mesenchymal markers, CTGF, fibronectin and YKL40 were determined using qPCR **(D)** or western blot assay **(E)**. The results are representative of three different experiments that gave similar results. Cell migration was assayed using a transwell chamber containing polycarbonate membrane inserts of 8-μm pore size (Corning, Costar) with fibronectin coating as described in the methods. The HF2355 GSCs transfected with a con siRNA or silenced for TALNEC2 were analyzed in this assay. The results are shown are the mean ± SE of triplicate experiments **(F)** (P<0.01). The HF2355 **(G)** and HF2414 **(H)** GSCs transfected with control or TALNEC2 siRNAs were irradiated (3 Gy) and self-renewal was analyzed after 2 weeks **(G, H)**. Kaplan-Meier survival curves for mice transplanted with the HF2587 GSCs transfected with a control siRNA or TALNEC2 siRNA1 (n = 11) were determined by both log-rank (Mantel-Cox) test and Gehan-Breslow-Wilcoxon test **(I)**.

Since TALNEC2 is expressed in high levels in GSCs, we examined the effects of its silencing on the self-renewal of these cells and on their stemness markers. We found that silencing of TALNEC2 in the HF2355 and HF2414 GSCs (Figure 5A) decreased the self-renewal (Figure 5B) and the frequency of sphere formation (Supplementary Figure 8). In addition, silencing of TALNEC2 decreased the expression of the stemness markers Nanog, SOX2 and Oct4 (Figure 5C, 5E), and the expression of the mesenchymal markers CTGF, fibronectin and YKL40 in the GSCs (Figure 5D, 5E).

Mesenchymal transformation of glioma is associated with increased cell infiltration [15, 35, 36]. In accordance with the effect of TALNEC2 on the expression of mesenchymal markers and with its increased expression in the mesenchymal GBM subtype, we found that silencing of TALNEC2 inhibited the migration of GSCs as indicated by transwell migration assays (Figure 5F). These assays were performed after 48 h of silencing. An equal number of cells were plated in transwell plates, and migration was determined after 6 h to exclude effects resulting from growth inhibition.

Mesenchymal transformation of GSC is also associated with resistance to radiotherapy. We found that silencing of TALNEC2 not only decreased the self-renewal of the GSCs but also increased their sensitivity to γ-radiation. Thus, irradiation of the cells (3 Gy) exerted a small effect on the self-renewal of the cells, whereas similar treatment of silenced cells decreased the self-renewal of the cells by about 80% (Figures 5G, 5H).

Since TALNEC2 expression was associated with GBM patient survival, we examined the effect of TALNEC2 silencing on the survival of mice bearing GSC-derived xenografts. For these experiments, we employed GSCs derived from GBM of a short-term survival patient. Mice bearing xenografts that were generated from these GSCs (HF2587) exhibited short-term survival of about 49 days. For these experiments, we employed GSCs that were silenced for TALNEC2 or treated with a control siRNA for 2 days to validate efficient silencing. Cells were implanted intracranially as described [37] and animal survival was determined. As presented in Figure 5I, silencing of TALNEC2 prolonged mouse median survival by 12 days (P<0.0001).

### Silencing of TALNEC2 induces changes in miRNA expression in glioma cells

**Figure 6:**
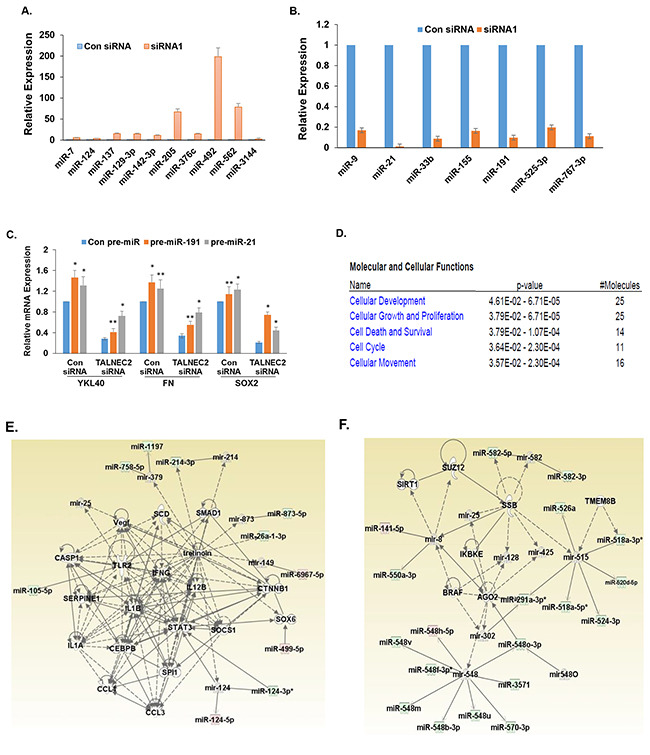
Alteration in miRNA expression in glioma cells silenced for TALNEC2 U87 cells were silenced for TALNEC2 using siRNA1 and siRNA2 for 2 days and the cells were analyzed for miRNA expression miRNome microRNA Profilers QuantiMir™. Validation for specific miRNAs that were altered in the TALNEC2 silenced U87 cells was performed for the HF2355 GSCs using RT-PCR. P<0.001 **(A, B)**. The role of miR-21 and miR-191 in TALNEC2 effects was examined by the transduction of TALNEC2 silenced HF2355 GSCS with lentivirus vectors expressing pre-miR-21 and pre-mir-191. The expression of stemness and mesenchymal markers was determined using RT-PCR **(C)**. Ingenuity functional clustering analysis **(D)** and generation of Ingenuity networks **(E, F)** were performed for the altered miRNAs in the TALNEC2-silenced glioma cells.

To explore the potential mechanisms that mediate TALNEC2 effects in glioma cells, we employed miRNA array analysis to compare control and TALNEC2-silenced glioma cells. We found that silencing of TALNEC2 in U87 cells resulted in an increased expression of miRNAs associated with tumor suppression [38, 39] (e.g., let-7b, miR-7, miR-124, miR-137, miR-129-3p, miR-142-3p, miR-205, miR-376c, miR-492, miR-562 and miR-3144) and in a decrease in the expression of miRNAs associated with tumor promotion [38–40] (e.g., miR-9, miR-21 miR-33b, miR-155, miR-191, miR-525-3p, and miR-767-3p). We validated the changes in the expression of some of these miRNAs in the HF2355 GSCs (Figure 6A, 6B) and the HF2414 GSCs (Supplementary Figure 9).

We further explored the role of miR-21, miR-191, miR-767 and miR-525 in TALNEC2 effects. Using lentivirus vectors overexpressing these specific pre-miRs we examined their ability to abrogate the inhibitory effects of TALNEC2 silencing on the mesenchymal transformation and stemness of GSCs. As presented in Figure 6C, overexpression of miR-21 or miR-191 modestly increased the expression of YKL40, fibronectin (FN) and SOX2. In contrast, overexpression of miR-21 and miR-191 in cells silenced for TALNEC2, partly rescued the decreased expression of the mesenchymal and stemness markers in the silenced cells, suggesting the decreased expression of these two miRs mediated some of effects observed in the TALNEC2-silenced cells. In contrast, overexpression of pre-miR-767 and pre-miR-525 did not alter the expression of any of these markers in the TALNEC2-silenced cells (data not shown).

Using bioinformatics tools, we further studied the functional associations of miRNAs that that showed at least a two-fold decrease in siRNA-treated cells relative to control, miRNAs that showed at least two-fold changes in siRNA-treated cells relative to control. Overall, we saw alteration in pathways of “Cellular Development” (25 miRNA), “Cellular Growth and Proliferation” (25 miRNA), “Cell Death and Survival (14 miRNAs), “Cell Cycle” (11 miRNA), and “Cellular Movement” (16 miRNA) (Figure 6D).

IPA generated seven networks each containing more than 10 miRNA-associated networks of interactions from the miRNA lists. The networks are associated with groups of classical oncogenes that play a pivotal role in the tumorigenic process in glioma such as PTEN, P53, ERK1/2, JNK, VEGF and caspase 1 (Figures 6E, 6F, Supplementary Figures 10A-10E) [41–43]. Looking at the altered miRNAs in the identified networks, we found that the top scoring networks contained transcription factors that are considered master regulators of mesenchymal transformation in glioma including CEBP/β, STST3 and RUNX1 (Figures 6E, Supplementary Figure 10B) [36], as well as a group of genes that are common to the EMT process including TGFβ1, SMAD1 and ZEB1 [44]. Another two interesting groups of genes that are altered in the TALNEC2-silenced cells are genes which play a role in miRNA biogenesis in the cytoplasm including Dicer1 and AGO2 (Figure 6F) [45] and a group of interleukins that regulate the fate of the macrophages/microglia (Supplementary Figure 10D), such as IL-12B, IL-1B, IL-1A and IL-23 [46, 47].

## DISCUSSION

GBMs, the most common and aggressive primary central nervous system (CNS) tumors, exhibit poor prognosis due to treatment resistance and infiltration of residual cancer tumor cells that are implicated in tumor recurrence [13, 48]. Long-noncoding RNAs are emerging as important regulators of various biological processes including cell-cycle progression and tumorigenesis [30, 49]. However, the specific lncRNAs in GBM and in the functions of GSCs are just beginning to be characterized.

In this study, we describe for the first time the identification of TALNEC2 as a putative E2F1-regulated lncRNA through a deep-sequencing analysis and demonstrate that TALNEC2 expression is regulated by activation of both ectopic and endogenous E2Fs. These transcription factors are best known for their involvement in the regulation of protein-coding genes required for cell-cycle progression, and particularly G1/S transition [50, 51]. TALNEC2 promoter contains a number of putative E2F1 binding sites and therefore is expected to be regulated by this transcription factor; however, additional studies are required to further characterize the exact mode of regulation. Additional lncRNAs, such as H19, ANRIL, ERIC, LINC00668, FAL1 and MA-linc1, have also been reported to be regulated by E2F1 [9–11, 52].

Our data indicate that consistently with a possible role in G1/S transition, the RNA levels of TALNEC2 increased as synchronized cells move through late G1 and early S. Importantly, its silencing, in a number of cancer cells lines, resulted in a significant increase in the number of cells in the G1 phase and a concomitant reduction in the number of cells in the S phase. Furthermore, upon silencing of TALNEC2, fewer cells were released from an arrest at the G1/S transition point and proceeded into S phase. In agreement with these effects on cell cycle distribution, silencing of TALNEC2 also inhibited cell proliferation.

Focusing on the expression and functions of TALNEC2 in GBM and glioma cells, we found that TALNEC2 was expressed in higher levels in GBM as compared to low-grade astrocytomas, oligodendroglioma and normal brain specimens. Moreover, TALNEC2 expression was the highest among IDHwt tumors compared to IDH-mutant tumors with the aggressive IDHwt groups (Classical-like, Mesenchymal-like, LGm6-GBM) showing higher mean levels than the less-aggressive IDHmut groups (CODEL, GCIMP-High) and in general, TALNEC2 showed higher expression in tumors with poorer prognosis.

These results were further strengthened by the RT-PCR data that validated the increased expression of TALNEC2 in GBM compared to low-grade tumors and normal brain specimens and in GBM derived from patients of short-term survival (9 months) as compared to patients with long-term survival (>36 months). Similarly, the expression of TALNEC2 was significantly increased in GSCs compared with human NSCs and in glioma cell lines compared with human astrocytes.

Non-coding RNAs (including both miRNAs and lncRNAs) have been recently implicated as important regulators of various tumorigenic processes including GBM [23, 25, 30]. Although most studies have explored the roles of miRNAs [40], recent reports implicated specific lncRNAs as critical factors in determining GBM growth, infiltration and response to therapies [23, 30]. Another E2F1-regulated lncRNA, H19, is significantly overexpressed in GBM tissues, its levels are associated with patient survival, and it was shown to promote invasion, angiogenesis, and stemness of GBM cells [53, 54]. The well-studied lncRNA HOTAIR is also upregulated in glioma cells, its expression is closely associated with glioma grade and poor prognosis and glioma cell growth is reduced following its depletion [23, 55]. Another lncRNA that is highly expressed in human glioma is CRNDE whose ectopic expression promotes glioma cell proliferation and migration both *in vitro* and *in vivo*. [56]. Expression of the tumor suppressor lncRNA MEG3 is markedly decreased in glioma tissues and its ectopic expression inhibits proliferation and promotes apoptosis in human glioma cell lines [57]. Additional lncRNAs that are implicated in glioma cell growth include XIST [58] and POU3F3 [59]. Recently, the expression of specific lncRNAs has also been associated with patient survival [23, 60, 61]. Our studies clearly demonstrate that TALNEC2 is associated with both aggressiveness and prognosis of GBM patients.

GBM contain a small subpopulation of self-renewing and highly tumorigenic cancer stem cells (GSCs) that contribute to therapy resistance and tumor recurrence and have been shown to be critical therapeutic targets [48, 62, 63]. Tumor recurrence and the survival of GBM patients have been mainly attributed to the GSC subpopulation that is present in the tumors [21, 48]. Importantly, we found that TALNEC2, in addition to being highly expressed in GSCs compared to NSCs and astrocytes, also regulated the stemness of these cells and that silencing of this lncRNA significantly decreased the self-renewal and stemness characteristics of the GSCs.

Epithelial-to-mesenchymal transition (EMT) is essential for the development of malignant carcinoma metastasis and drug resistance [64–67]. Recently, mesenchymal transformation has also been shown to occur in GBM [35, 67–69], and has been reported to be associated with the increased infiltration, acquisition of stemness characteristics and resistance to radiotherapy of GSCs [67, 70, 71]. We found that TLANEC2 regulated the mesenchymal transformation of GSCs and that silencing of TALNEC2 decreased both the migration and expression of mesenchymal markers in these cells. The mesenchymal transformation of GSCs has been reported to be mediated by various pathways associated with activation of STAT3, C/EBPβ, ZEB1, SNAIL and NF-kb and by various non-coding RNAs [36, 70, 71]. Although the expression and function of specific lncRNAs have been studied in various functions of glioma cells and GSCs [22, 27], the role of specific lncRNAs in the mesenchymal transformation of GSCs has not been reported. Altogether, our results indicate that silencing of TALNEC2 inhibited the proneural-to-mesenchymal transit of GSCs and the TCGA analysis of increased expression of TALNEC2 in the mesenchymal subtype of GBM further supports a role of TALNEC2 in this pathway.

One of the main factors contributing to therapy failure in GBM is the activation of specific signaling pathways in GSCs, which are associated with resistance to both chemo- and radiotherapy. Indeed, the mesenchymal transformation of GSCs has been associated with resistance to radiation [71]. We found that in parallel to the inhibition of the stemness and mesenchymal transformation of GSCs, silencing of TALNEC2 also enhanced the sensitivity of the cells to γ-irradiation.

GBM patients exhibit poor prognosis due to therapy resistance and increased infiltration of residual GSCs, which lead to tumor recurrence after resection [13, 17, 40]. To examine the role of TALNEC2 in the tumorigenic potential of GSCs *in vivo* we generated xenografts from two GSCs derived from GBM of short-term survival patients. We found that silencing of TALNEC2 expression in these GSCs significantly increased the mean survival of the xenograft-bearing mice. These findings further demonstrate that TALNEC2 silencing decreased the tumorigenic potential of GSCs *in vivo*, together with the decreased expression of TALNEC2 in long-term survival GBM patients, suggest that inhibiting this pathway may improve GBM patient prognosis. Since normal neural cells express significantly lower levels of TALNEC2, silencing of TALNEC2 in GSCs which decreases their self-renewal and mesenchymal transformation can be employed as a novel therapeutic approach to abrogate the oncogenic potential of GSCs with no damage to normal cells in the brain.

The mechanisms by which TALNEC2 mediates its effects are currently not understood. We performed microarray analysis of miRNA expression of glioma cells silenced for TALNEC2 and found that silencing of TALNEC2 increased the expression of several tumor suppressor miRNAs such as miR-137, miR-124, miR-205, miR-7 and miR-492, whereas it decreased the expression of some oncomiRs such as miR-21, miR-155, miR-33b and miR-191. The molecular mechanisms that underlie the effect of TALNEC2 on the levels of these microRNAs are currently unknown. Core-analysis of the altered miRNAs identified top scoring networks of interacting miRNA, which interplay through several genes and molecules known to be involved in cancer and EMT including STAT3, CEBP/β, RUNX1, VEGF and TGF-β [36, 70, 71]. These findings are in accordance with our results demonstrating an important role of TALNEC2 in modulating the stemness, mesenchymal transformation and radiation resistance of glioma cells and GSCs. Rescue experiments overexpressing specific pre-miRs that were downregulated in the silenced cells demonstrated that miR-21 and miR-191, which have been associated with the control of GSC stemness and the EMT process [72, 73], were partly associated with TALNEC2 effects on the mesenchymal transformation and stemness of GSCs. Additional functions of TALNEC2 such as association with specific miRNAs and proteins are currently being studied.

In summary, our results indicate that TALNEC2 acts as an important marker for GBM and GSC aggressiveness and as a prognostic marker for GBM patient survival. Moreover, TALNEC2 plays a role as a major factor in controlling the growth, stemness and mesenchymal transformation of GSCs. The expression of TALNEC2 in a wide number of tumors and the effects of its silencing on the proliferation and cell-cycle progression of cancer cells derived from other tissues including breast and lung strongly suggest that this lncRNA might play a role in the development and progression of other tumors, in addition to GBM.

## MATERIALs AND METHODS

### Cell cultures

The glioma cell lines A172, and U87 were obtained from the American Type Culture Collection (Manassas, VA). Cells were maintained as previously described [32]. U2OS human osteosarcoma cells were grown in Dulbecco's modified Eagle's medium (DMEM) supplemented with 5% fetal bovine serum (FBS). SAOS-2 human osteosarcoma cells were grown in Dulbecco's modified Eagle's medium (DMEM) supplemented with 10% FBS. WI38 human embryonic lung fibroblasts were grown in minimal essential medium (MEM) supplemented with 10% FBS, 2mM L-glutamine, 1mM sodium pyruvate and non-essential amino acids. H1299 human lung adenocarcinoma cells were grown in RPMI 1640 medium supplemented with 5% FBS. MCF7 breast cancer cells were grown in DMEM supplemented with 10% FBS. Cells were maintained at 37 °C in a humidified atmosphere containing 8% CO2. To induce activation of ER-E2F1, cells were treated with 100 nM of 4-hydroxytamoxifen (OHT, Sigma-Aldrich, St. Louis, MO) for the times indicated. Where indicated, cycloheximide (Sigma) was used for 8 h at 10 μg/ml. Hydroxyurea (sigma) was used at 2 mM for 18 h.

### Generation of primary GSC cultures

All human materials were used in accordance with the policies of the Institutional Review Board at Henry Ford Hospital. Generation of GSCs from fresh GBM specimens was performed as previously described [33]. The GSCs were examined for the expression of CD44, Bmi-1, CD133, Sox2 and nestin, self-renewal, expression of astrocytic, oligodendrocytic and neuronal markers upon differentiation, and for their tumorigenic potential in nude mice [74].

### Neurosphere formation assay

The ability of GSCs to form secondary neurospheres was determined as previously described [32, 33, 54]. Briefly, disaggregated cells were subjected to the appropriate treatments, and cells were plated onto 24-well plates at a density of 10 or 100 cells/well through limiting dilutions. The number of neurospheres per well was determined 10 or 14 days thereafter for 8 different wells. Spheres that contained more than 20 cells were scored. Results are presented as percentages of maximal neurospheres formed in control untreated cells.

### *In vitro* limiting dilution assay

GSCs were plated in 96-well plates in decreasing numbers per well (50, 20, 10, 5, 2 and 1) as recently described [54]. Ten days later the generation and number of neurospheres were quantified in each well. Extreme limiting dilution analysis was performed using software available at http://bioinf.wehi.edu.au/software/elda.

### Small interfering RNA transfection

Small interfering RNA (siRNA) duplexes were synthesized and purified by Dharmacon (Lafayette, CO). The siRNA sequences for targeting TALNEC2 mRNA were siRNA1: CCAAAGGCCCTGAAGTACACAGTTT and siRNA2: AGCAGTGTATTAGAAGACAACTGAA. Transfection of siRNAs was done using Oligofectamine (Invitrogen, Carlsbad, CA) according to the manufacturer's instructions. Experiments were performed 48 h after transfection.

### Western blot analysis

Cell pellet preparation and Western blot analysis were performed as previously described [75].

### Transwell migration assay

Transwell chambers (BD Biosciences, San Jose, CA) were used for analyzing cell migration as recently described [75].

### Real-time PCR

Total RNA was extracted using RNeasy midi kit according to the manufacturer's instructions (Qiagen, Valencia, CA). Reverse transcription reaction was carried out using 2 μg total RNA as described for the RT-PCR analysis. A primer optimization step was tested for each set of primers to determine the optimal primer concentrations. Primers, 25 μL of 2x SYBR Green Master Mix (Invitrogen), and 30 to 100 ng cDNA samples were resuspended in a total volume of 50 μL PCR amplification solution. The following primers were used:

FN- forward TGGCCAGTCCTACAACCAGT,

reverse CGGGAATCTTCTCTGTCAGC;

α-SMA-forward CCGACCGAATGCAGAAGGA,

reverse ACAGAGTATTTGCGCTCCGAA;

YKL-40 forward TGCCCTTGACCGCTCCTCT GTACC,

reverse GAGCGTCACATCATTCCACTC;

olig2-forward CAAATCTAATTCACATTCGGAA GGTTG,

reverse GACGATGGGCGACTAGACACC

CTGF-forward GGGAAATGCTGCGAGGAGT,

reverse AGGTCTTGGAACAGGCGCTC;

Oct4 - forward ATCAGCCACATCGCCCAGCA,

reverse CCCAGCAGCCTCAAAATCCT;

Sox2-forward TGGGTTCGGTGGTCAAGTC,

reverse CGCTCTGGTAGTGCTGGGA;

S12-forward, TGCTGGAGGTGTAATGGACG,

reverse CAAGCACACAAAGATGGGCT.

Reactions were run on an ABI Prism 7000 Sequence Detection System (Applied Biosystems, Foster City, CA). Cycle threshold (Ct) values were obtained from the ABI 7000 software. S12 or ß-actin levelswere also determined for each RNA sample as controls.

### Subcellular localization of TALNEC2

RNA was extracted from nucleus and cytoplasm as previously described using the Invitrogen nuclear extraction protocol [11]. Briefly, cells were incubated in 0.5 ml of hypotonic buffer for 15 minutes on ice, 10% NP40 was then added and the homogenate was centrifuged for 10 min at 3,000 rpm at 4°C. The RNA from nuclear fraction (pellet) was extracted by the TRI Reagent and RNA from cytoplasmic fraction (supernatant), using the Phenol-Chloroform method. RNA levels of the nuclear and the cytoplasmic fractions were analyzed by RT-PCR and were normalized to levels of external RNA.

### TCGA analysis

LncRNA data were downloaded for LGG and GBM cases from the lncRNAtor online tool, using the differential expression browser (http://lncrnator.ewha.ac.kr/expression.htm, 20 April, 2016). Clinical data were taken from the pan-glioma analysis from TCGA (Supplementary Table 1; https://tcga-data.nci.nih.gov/docs/publications/lgggbm_2016/, 20April,2016). FPKM data for LINC00116 was extracted from the data matrices for 205 primary lower grade glioma (LGG) and 136 primary GBM cases. One-way ANOVA, followed by post-hoc t-tests, is used to test for differences in mean expression between sample classes. Comparisons are visualized by boxplots (log2 scale).

Kaplan-Meier survival estimates were used to draw graphs of overall survival. Log-rank tests assessed differences in the expected survival experience between patient groups. Here patients are grouped by TALNEC2 expression quartiles with quartile 1 expressing the lowest TALNEC2 levels.

### Global miRNA expression

U87 glioma cells transfected with a control (N=2) or TALNEC2 siRNA sequence (N=2) and miRNA analysis was erformed using miRNome microRNA Profilers QuantiMir™ (92, Qiagen) according to the manufacturer's instructions. Expression level of the miRNAs was normalized to an average of 3 internal short RNA controls (Human U6 snRNA, RNU43 snoRNA, Hm U1 snRNA ) using the comparative CT method for relative quantification. There were 1110 miRNA targeted in the miRNA assay. Of these, 93 were not measurable in either sample and were therefore excluded from analysis. To assess the difference in miRNA expression, we used the log2-ratio of expression in treated cells relative to expression in control cells. We found that of the 1017 measured miRNAs, 382 showed at least a 2-fold change. This included 245 down-regulated and 137 upregulated miRNAs in the siRNA treated cells relative to untreated control cells.

### Ingenuity pathway analysis

To attribute functional changes to the deregulation of miRNA in the presence of the siRNA, relative to the control, we used Ingenuity Pathway Analysis software. The 1017 measured miRNA and 59 measured mRNA were each uploaded. All mRNA and 96% of the miRNA (981/1017) could be mapped into the IPA database. Core analysis was performed on the miRNA data to assess functional sets of altered miRNA and to generate networks of altered molecules. The full set of measured miRNA was used as a reference to determine if the changed sets of miRNA were represented more frequently than expected in each functional group using Fisher's exact tests. MiRNA target prediction was used to identify inversely altered miRNA and mRNA, signifying concordant change in response to TALNEC2 silencing. Expression values for these miRNA and mRNA were then entered into core analysis to identify networks of interrelated molecules.

### Public lncRNA dataset

Normalized gene expression data from a prior study of long non-coding RNA [2] were downloaded from the Gene Expression Omnibus archive (GEO, GSE28866, April 2, 2016). Expression for TALNEC2 (ID: “uc002ukz.1”) was identified by mapping the hg18 coordinates provided in the GEO dataset (chr2:177202809-177202992-) to GRCh38/h38 coordinates (chr2:176629835-176630018-) using the UCSC Genome Browser LiftOver tool (last accessed April 2, 2016). This region overlays the TALNEC2 region (chr2: 176629589-176637931-; GRCh38.p5) according to ensemble (http://useast.ensembl.org/Homo_sapiens/Gene/Summary?db=core;g=ENSG00000163364, last accessed April 2, 2016). Barplots of normalized expression levels per assayed cell line from cancer tissues, normal adult tissues, and normal fetal tissues, were constructed to visualize the data.

### GSC-derived xenografts

Following the guidelines of Henry Ford Hospital's IACUC, dissociated GBM neurospheres were inoculated intracranially in nude mice (Nu/Nu) as described previously [37, 54, 67]. Briefly, animals were anesthetized and a Hamilton syringe was used to inject the HF2587 GSCs transfected TALNEC2 or control siRNAs through a 3-mm hole to the right of the bregma, at a depth of 2.5 mm, at a rate of 0.5 μL/30 s. The surgical zone was flushed with sterile saline, the hole sealed with bone wax, and the skin over the injection site sutured. Animals were monitored daily and sacrificed at the end of the experiment.

### Statistical analysis

Quantitative measures are presented as the mean values ± SD and visualized in bar charts. The data of patient specimens are presented in scatterplots with mean and standard error of the mean noted. Data were analyzed using ANOVA or a Student's t-test with correction for data sets with unequal variances. Fold-change data were analyzed on a log 2 scale as appropriate. Kaplan-Meier analysis was used to produce survival curves with differences tested between groups by the log-rank test.

## SUPPLEMENTARY MATERIALS FIGURES


