# Correction: The novel long non-coding RNA TALNEC2, regulates tumor cell growth and the stemness and radiation response of glioma stem cells

**DOI:** 10.18632/oncotarget.27383

**Published:** 2021-12-21

**Authors:** Shlomit Brodie, Hae Kyung Lee, Wei Jiang, Simona Cazacu, Cunli Xiang, Laila M. Poisson, Indrani Datta, Steve Kalkanis, Doron Ginsberg, Chaya Brodie

**Affiliations:** ^1^ Everard and Mina Goodman Faculty of Life Sciences, Bar-Ilan University, Ramat-Gan, Israel; ^2^ Davidson Laboratory of Cell Signaling and Tumorigenesis, Hermelin Brain Tumor Center, Department of Neurosurgery, Detroit, MI, USA; ^3^ Department of Public Health Sciences, Center for Bioinformatics, Henry Ford Hospital, Detroit, MI, USA

**This article has been corrected:** The bioinformatics analysis of TCGA data in Figure 4 (panels A, B, C, I) was inadvertently conducted on LINC00116. We have reworked that analysis using the LINC01116 expression values so that it is aligned with the rest of the paper, which discusses LINC01116. The redrawn Figures 4A-C and 4I and edits to the associated paragraph in the text are shown below. Please note that similar trends are shown for LINC00116 and LINC01116 so the primary message of the paper is not affected. The authors declare that these corrections do not change the results or conclusions of this paper.

Original article: Oncotarget. 2017; 8:31785–31801. 31785-31801. https://doi.org/10.18632/oncotarget.15991

**Figure 4:**
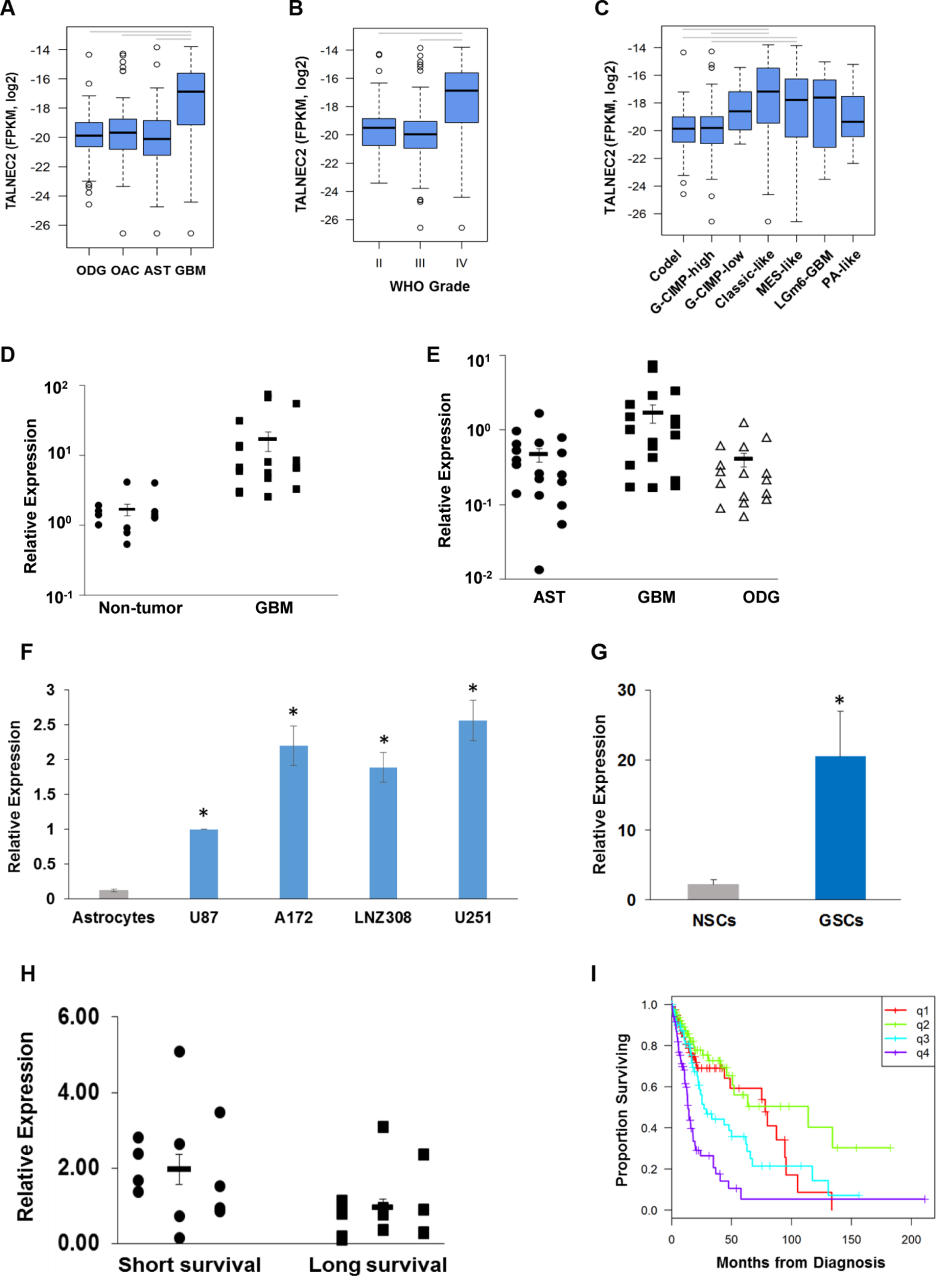
Expression of TALNEC2 in GBM, glioma cell lines and GSCs.

Expression of TALNEC2 in glioma tissues was determined from RNA-sequencing data from The Cancer Genome Atlas project. The distribution of expression is shown by boxplots for histology (**A**), WHO grade (**B**), and supervised IDH-methylation classes (**C**). Average expression was compared by ANOVA (P<0.0001) with follow-up t-tests (P<0.05, grey bars). Total RNA was extracted from normal brains (Non-tumor), astrocytoma (AST), oligodendroglioma (ODG) and GBM specimens and the expression of TALNEC2 was determined using real-time PCR (**D**, **E**). Results are normalized relative to the levels of S12 mRNA and are presented relative to a reference sample as dot-plots around the estimate of the mean and SEM bars. GBM has higher TALNEC2 expression, on average, than non-tumor brain, AST, or ODG (P<0.01). The mean expression of TALNEC2, measured using real-time PCR, in glioma cell lines relative to human astrocytes (**F**) is shown by barplots with SEM bars (student test P<0.001 indicated by *). Twelve different primary GSCs, generated from GBM specimens, show increased mean expression of TALNEC2 (by RT-PCR), relative to human NSCs (**G**, P<0.001). GBM specimens obtained from sort-term survivors (<9 months, n=13) have higher mean TALNEC2 expression compared to long-term survivors (>36 months, n=12; P< 0.01, **H**). Kaplan-Meier survival estimates of overall survival are plotted for patients grouped by TALNEC2 expression quartiles (q1=lowest expression; log-rank p= 0.000064, I). The results are presented as the mean values ± SD. Data were analyzed using analysis of variance or a Student's t-test.

