# Supplementary material for: The novel long non-coding RNA TALNEC2, regulates tumor cell growth and the stemness and radiation response of glioma stem cells
Source: Oncotarget. 2017 Mar 7;8(19):31785–801. doi: 10.18632/oncotarget.15991 (PMC5458248; doi:10.18632/oncotarget.15991)
Supplement: Supplementary file 1 [file oncotarget-08-31785-s001.pdf]

The novel long non-coding RNA TALNEC2, regulates tumor cell growth and the stemness and radiation response of glioma stem cells

SUPPLEMENTARY FIGURES

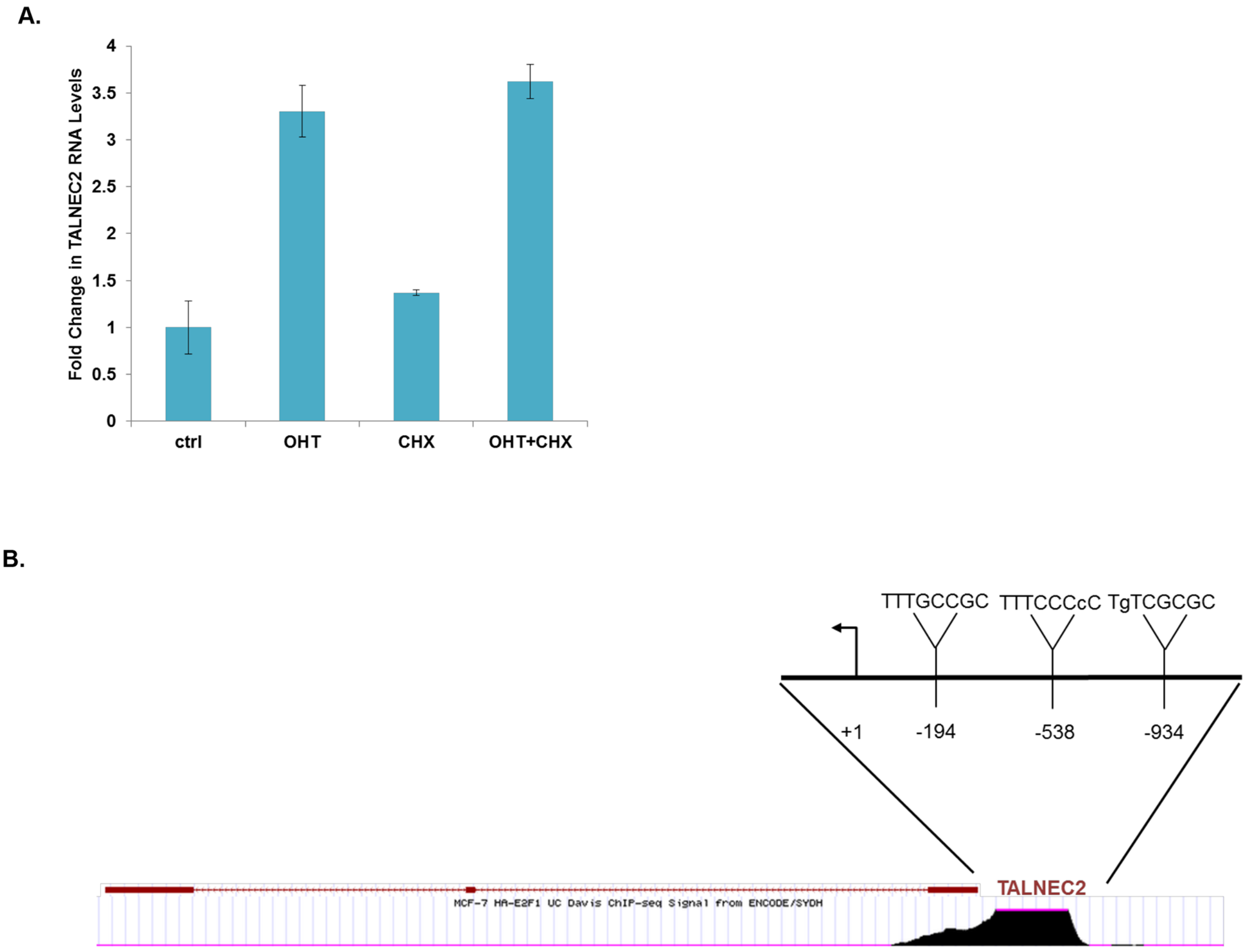

Supplementary Figure 1: Regulation of TALNEC2 by E2F. U2OS cells expressing ER-E2F1 were left untreated or incubated with OHT (100 nM) for 24 h, then cells were treated or not with 10 µg/mL cycloheximide (CHX) for 8 h. RNA was extracted and TALNEC2 RNA levels were determined by real-time RT-PCR and normalized to GAPDH levels. Real-time PCR experiments were performed in triplicates **(A)**. Schematic representation of the TALNEC2 promoter. The E2F-binding sites are represented as 8-mer nucleotide sequences. The transcription start site (+1) is indicated by an arrow. Schematic representation of human chromosome 2 at the TALNEC2 locus. MCF-7 Chip-seq published data from UCSC Genome Browser ENCODE transcription factor binding tracks is presented (HA-E2F1 antibodies were used) **(B)**.

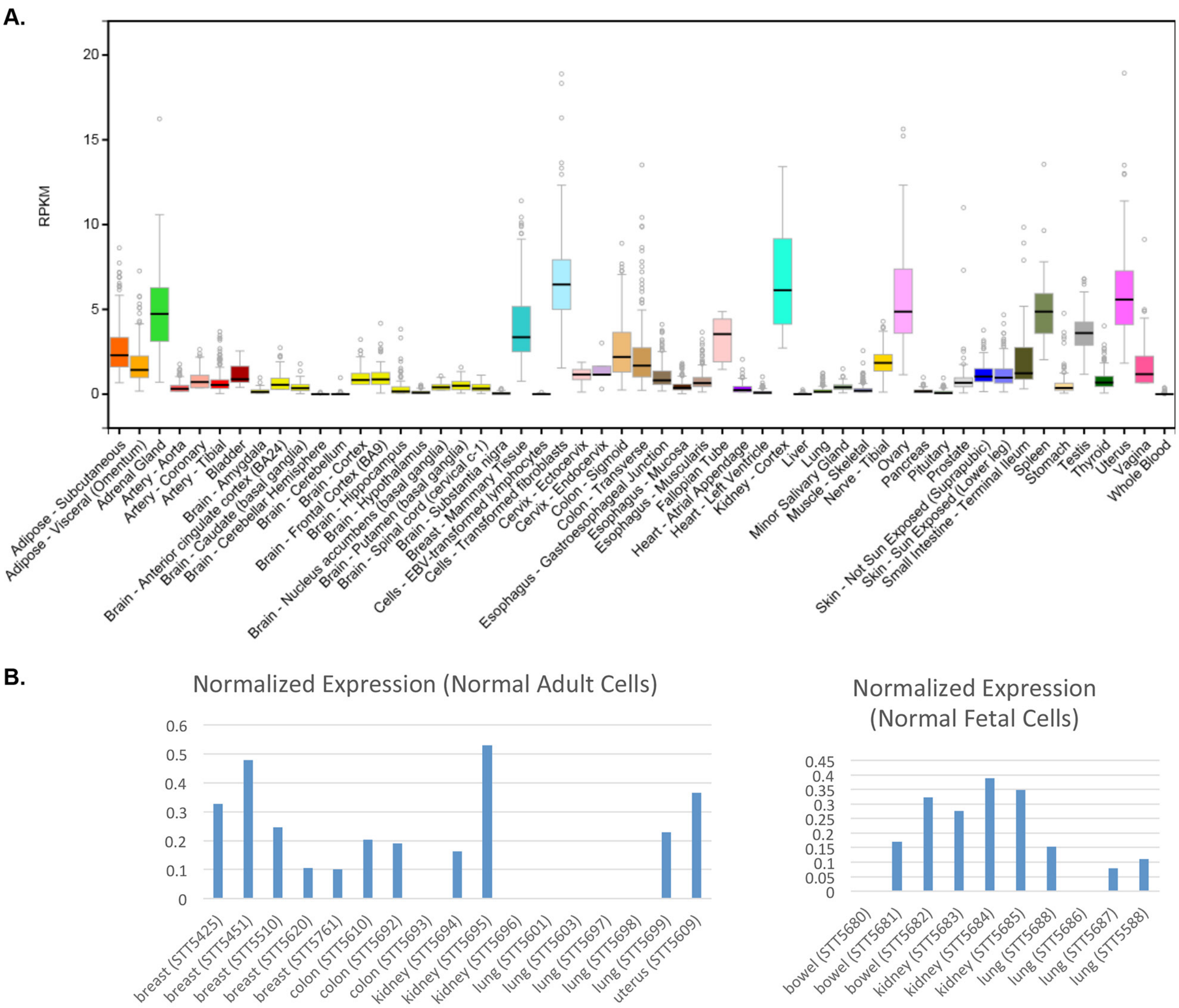

Supplementary Figure 2: Expression of TALNEC2 in various tissues and cells. Barplots are drawn of the normalized expression levels for TALNEC2 normal adult and normal fetal tissues. These data were obtained from a prior study of long non-coding RNAs available through the Gene Expression Omnibus archive (GEO, GSE28866, April 2, 2016). Expression for TALNEC2 (ID: "uc002ukz.1") was identified by mapping the hg18 coordinates provided in the GEO dataset (chr2:177202809-177202992-) to GRCh38/h38 coordinates (chr2:176629835-176630018-) using the UCSC Genome Browser LiftOver tool (last accessed April 2, 2016). This region overlays the TALNEC2 region (chr2: 176629589-176637931-; GRCh38.p5) according to ensemble (http://useast.ensembl.org/Homo_sapiens/Gene/Summary?db=core;g=ENSG00000163364, last accessed April 2, 2016).

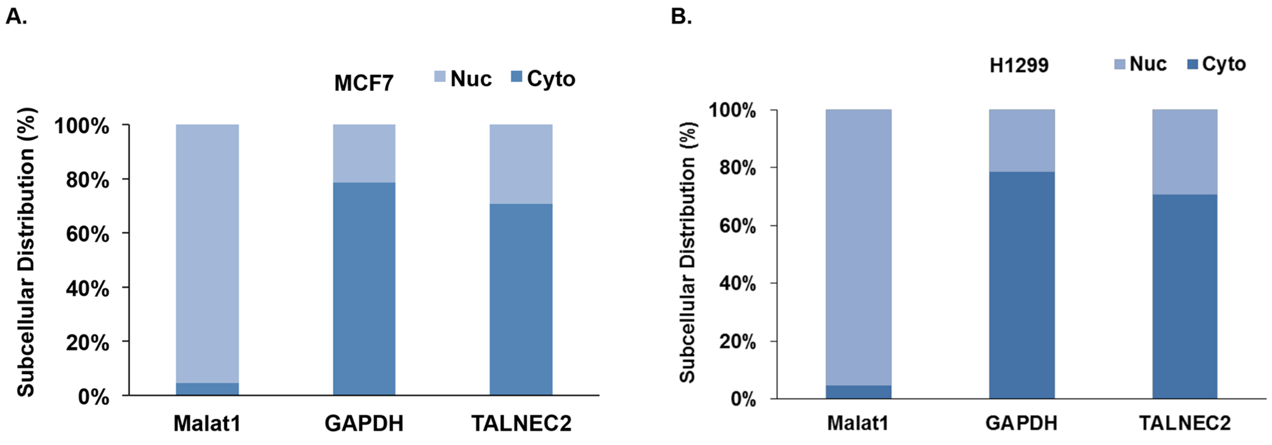

Supplementary Figure 3: TALNEC2 is a cytosolic lncRNA that regulates cell cycle progression. RNA was extracted from nucleus and cytoplasmic fractions of the MCF-7 **(A)** and H1299 **(B)** cells and the levels of nuclear control transcript (MALAT1), cytoplasmic control transcript (GAPDH or Tubulin), and TALNEC2 were determined by Real-time PCR in nuclear and cytoplasmic fractions and normalized to levels of external RNA. Graph presented as fraction of subcellular enrichment out of whole cell levels. Real-time PCR experiments were performed in triplicate **(A, B)**.

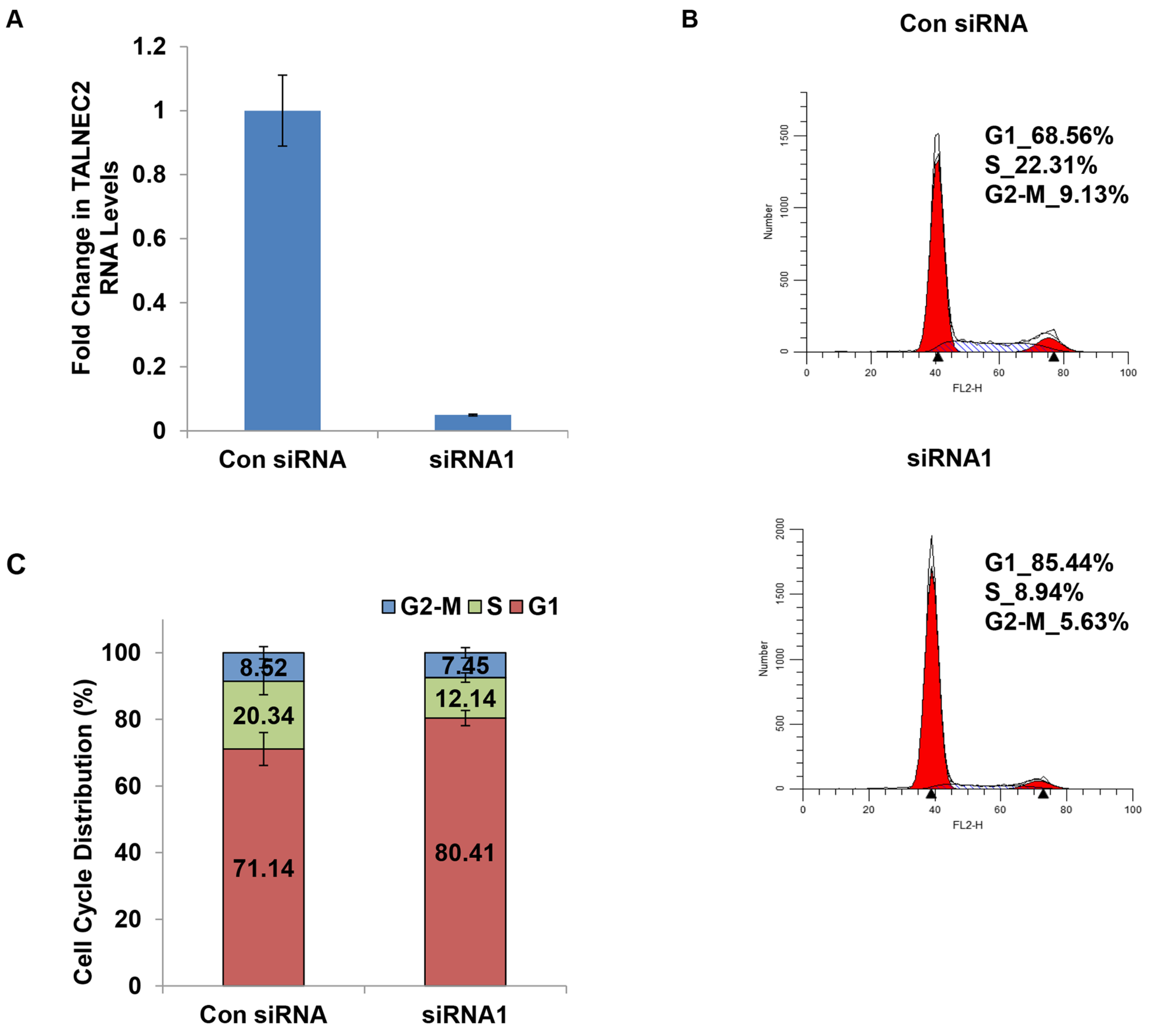

Supplementary Figure 4: Silencing of TALNEC2 arrests cells in the G1 phase of the cell cycle. H1299 cells were transfected with either a control siRNA (Con siRNA, 100nM) or siRNAs directed against TALNEC2 (siRNA1 (25nM), siRNA2 (100nM)). Cells were harvested 72 hours post transfection. RNA was extracted and TALNEC2 RNA levels were determined by real-time PCR and normalized to GAPDH levels. Real-time PCR experiments were performed in triplicates **(A)**. Cells were analyzed by FACS using propidium-iodide (PI) protocol. Percentages of cells in G1, S, and G2/M cell-cycle phases are shown **(B+C)**. The results are either representative **(B)** or average of 3 independent experiments **(C)**.

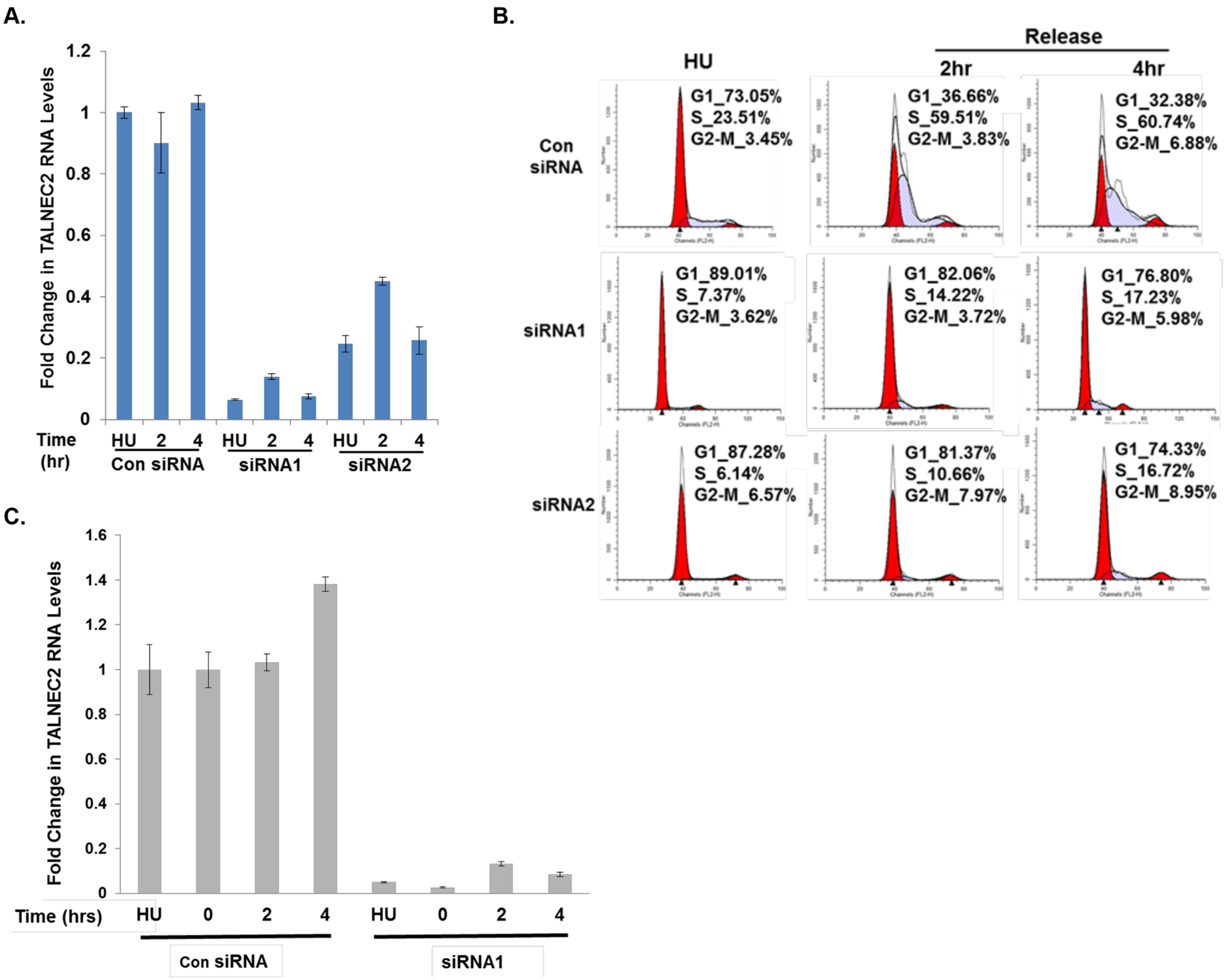

Supplementary Figure 5: Role of TALNEC2 in cell cycle progression. MCF-7 cells were transfected with either a nonspecific siRNA (Con siRNA, 100nM) or siRNAs directed against TALNEC2 (siRNA1 (25nM), siRNA2 (100nM)). Next, cells were incubated with hydroxyurea (2mM) for 18 hours. 72 hours post transfection cells were harvested or allowed to resume growth by fresh media wash and growth in the fresh media for times indicated. RNA was extracted and TALNEC2 RNA levels were determined by real-time PCR and normalized to GAPDH levels. Real-time PCR experiments were performed in triplicates **(A)**. Cells were analyzed by FACS using propidium-iodide (PI) protocol. Percentages of cells in G1, S, and G2-M cell-cycle phases are depicted **(B)**. H1299 cells were transfected with either a nonspecific siRNA (Con siRNA, 100nM) or siRNAs directed against TALNEC2 (siRNA1 (25nM), Next, cells were incubated with hydroxyurea (2mM) for 18 hours. 72 hours post transfection cells were harvested or allowed to resume growth by fresh media wash and growth in the fresh media for times indicated. RNA was extracted and TALNEC2 RNA levels were determined by real-time PCR and normalized to GAPDH levels. Real-time PCR experiments were performed in triplicates **(C)**.

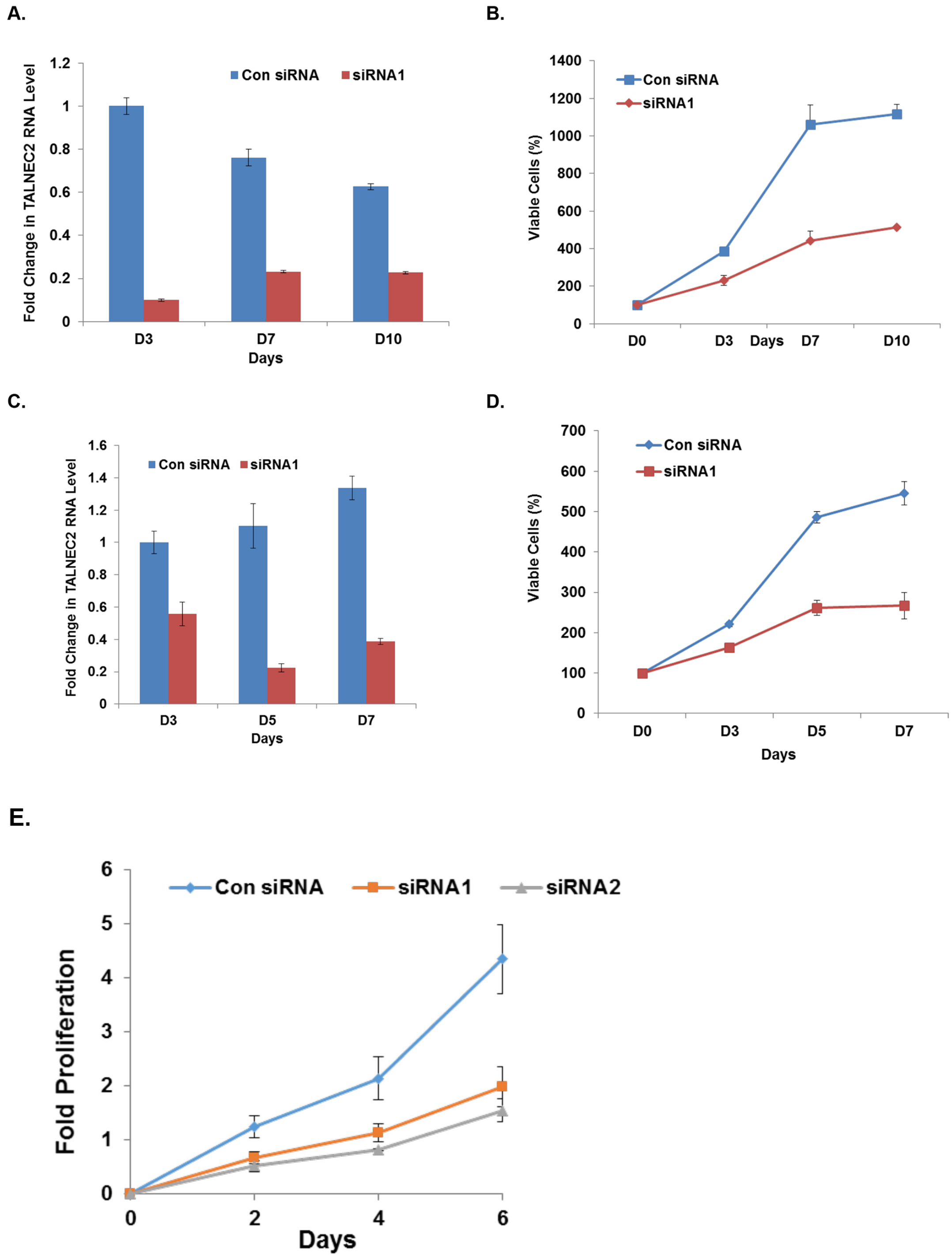

Supplementary Figure 6: Silencing of TALNEC2 inhibits cell proliferation. MCF-7 cells were transfected with either a nonspecific siRNA (Con siRNA, 25nM) or siRNA directed against TALNEC2 (siRNA1, 25nM). Cells were left untreated and were harvested for indicated days post transfection. RNA was extracted and TALNEC2 RNA levels were determined by Real-time PCR and normalized to GAPDH levels. Real-time PCR experiments were performed in triplicates **(A, C)**. Cell proliferation was measured by MTT assay **(B, D)**. D0 represents day prior to transfection analyzed for number of cells seeded. For the A172 glioma cells, cells were silenced with siRNA1 and siRNA2. Levels of TALNEC2 were determined after 3 days using RT-PCR and cell proliferation was determined at the indicated time points using MTT **(E)**. The results are representative of three different experiments that gave similar results.*P<0.01.

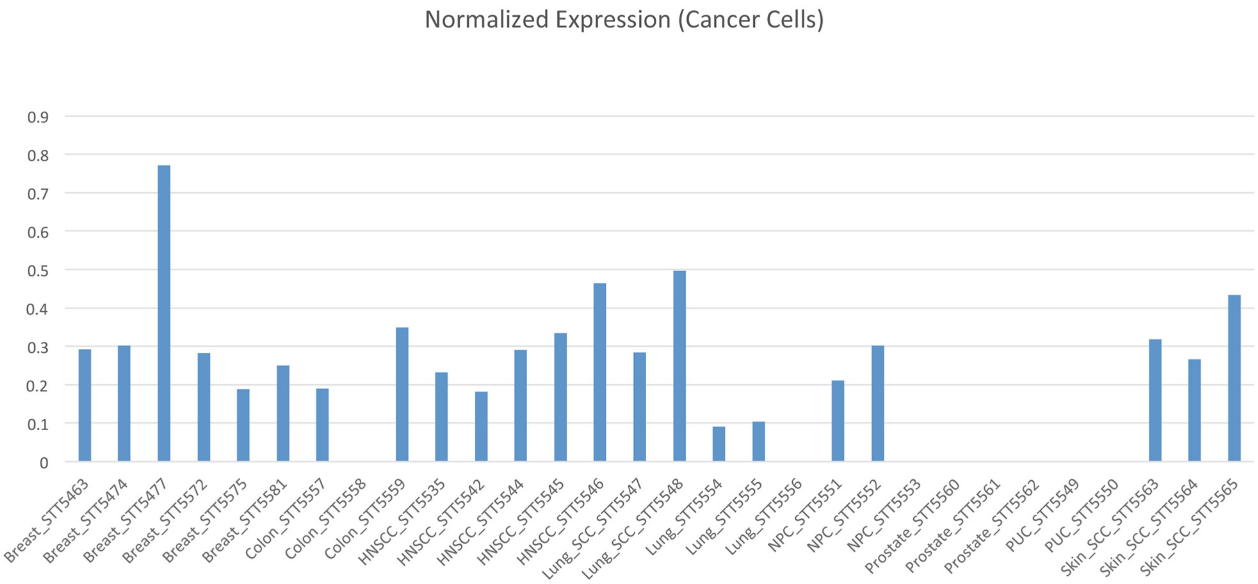

Supplementary Figure 7: Expression of TALNEC2 in cell lines derived from cancer tissues. Expression was determined as described in Supplementary Figure 2.

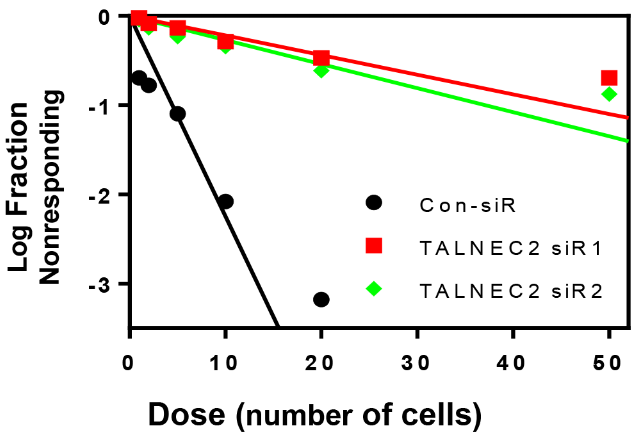

Supplementary Figure 8: The effect of TALNEC2 on the self-renewal of GSCs. *In vitro* extreme limiting dilution assay (ELDA) demonstrated that silencing of TALNEC2 in the HF2414 GSCs decreased the frequency of neurosphere formation.

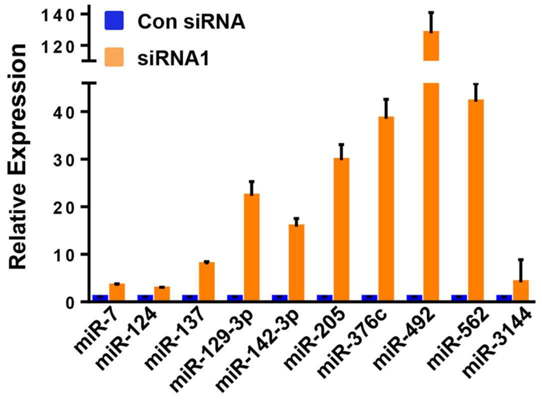

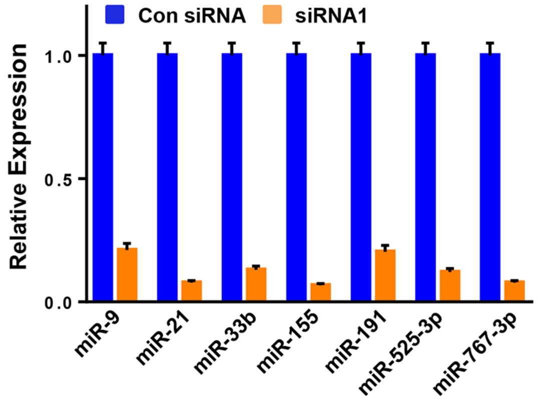

Supplementary Figure 9: Validation of miR array results in TALNEC2-silenced GSCs. Validation for specific miRNAs that were altered in the TALNEC2 silenced U87 cells was performed in the HF2414 GSCs using RT-PCR. P<0.001.

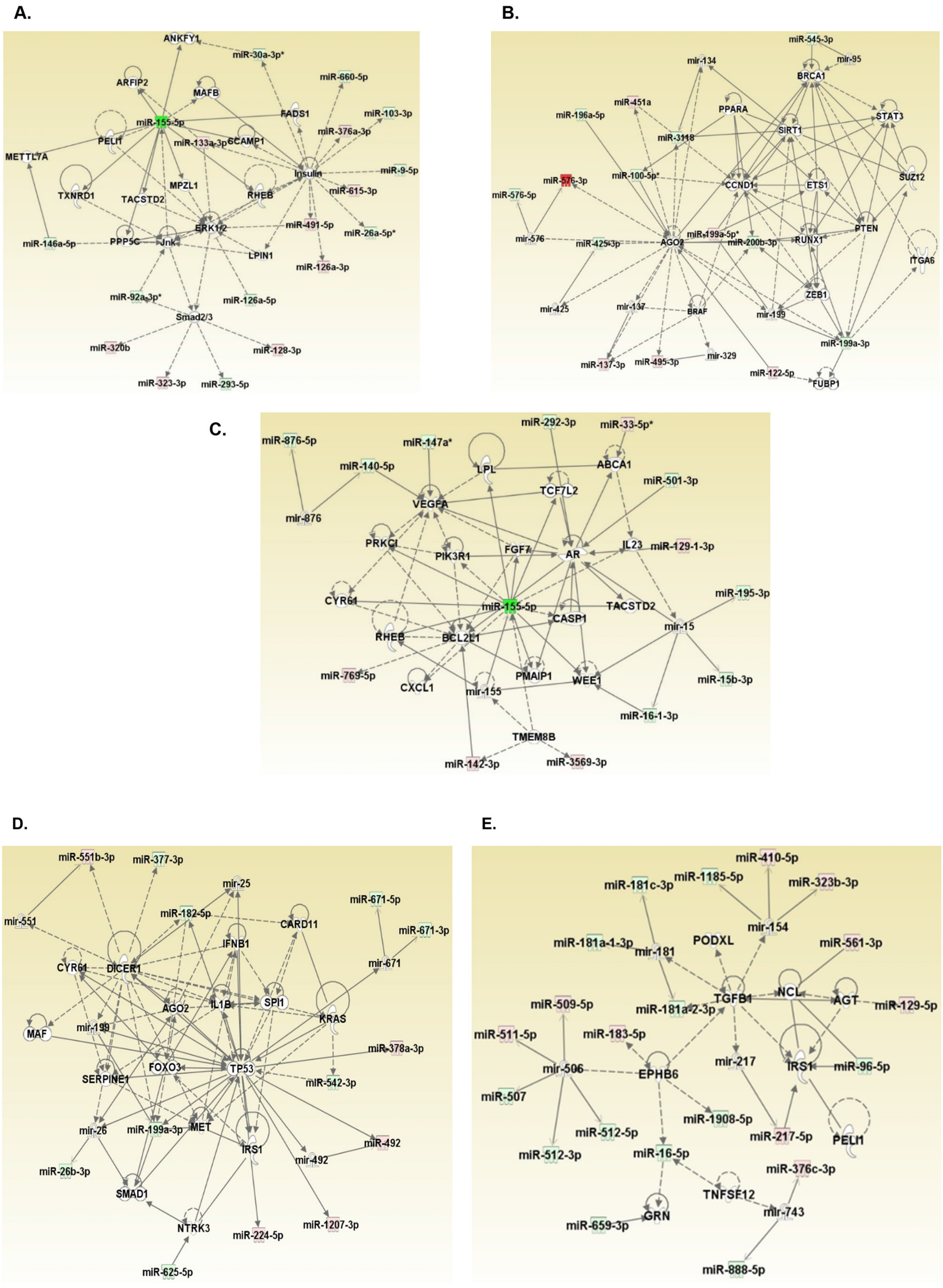

Supplementary Figure 10: IPA for miRNAs altered in TALNEC2-silenced glioma cells. Ingenuity functional analysis and generation of Ingenuity networks **(A-E)** were performed for the altered miRNAs in the TALNEC2-silenced glioma cells.
